# Mid-term outcomes of laparoscopic vaginal stump-round (Kakinuma method) and stump-uterosacral (Shull method) ligament fixation for pelvic organ prolapse: A retrospective comparative study

**DOI:** 10.1186/s12893-024-02429-9

**Published:** 2024-05-06

**Authors:** Toshiyuki Kakinuma, Kaoru Kakinuma, Kyouhei Ueyama, Takumi Shinohara, Rora Okamoto, Ken Imai, Nobuhiro Takeshima, Kaoru Yanagida, Michitaka Ohwada

**Affiliations:** Department of Obstetrics and Gynecology, International University of Health and Welfare Hospital, 537-3, Iguchi, Nasushiobara-City, 329-2763 Tochigi Japan

**Keywords:** Pelvic organ prolapse, Native tissue repair, Laparoscopic surgery, Round ligament, Kakinuma method, Shull method

## Abstract

**Background:**

Laparoscopic sacrocolpopexy (LSC) and robot-assisted sacrocolpopexy (RSC) using mesh are popular approaches for treating pelvic organ prolapse (POP). However, it is not uncommon that native tissue repair (NTR) should be presented as an option to patients who are expected to have extensive intraperitoneal adhesion or patients for whom LSC or RSC is difficult owing to various risk factors. Laparoscopic vaginal stump–uterosacral ligament fixation (Shull method) has been introduced as a method for NTR in case of POP. However, effective repair using this surgical procedure may not be possible in severe POPs. To solve the problems of the Shull method, we devised the laparoscopic vaginal stump–round ligament fixation (Kakinuma method) in which the vaginal stump is fixed to the uterine round ligament, a histologically strong tissue positioned anatomically higher than the uterosacral ligament. This study aimed to retrospectively and clinically compare the two methods.

**Methods:**

Of the 78 patients who underwent surgery for POP between January 2017 and June 2022 and postoperative follow-up for at least a year, 40 patients who underwent the Shull method (Shull group) and 38 who underwent the Kakinuma method (Kakinuma group) were retrospectively analyzed.

**Results:**

No significant differences were observed between the two groups in patient background variables such as mean age, parity, body mass index, and POP-Q stage. The mean operative duration and mean blood loss in the Shull group were 140.5 ± 31.7 min and 91.3 ± 96.3 ml, respectively, whereas the respective values in the Kakinuma group were 112.2 ± 25.3 min and 31.4 ± 47.7 ml, respectively. Thus, compared with the Shull group, the operative duration was significantly shorter (*P* < 0.001) and blood loss was significantly less (*P* = 0.003) in the Kakinuma group. Recurrence was observed in six patients (15.0%) in the Shull group and two patients (5.3%) in the Kakinuma group. Hence, compared with the Shull group, recurrence was significantly less in the Kakinuma group (*P* = 0.015). No patients experienced perioperative complications in either group.

**Conclusions:**

The results suggest that the Kakinuma method can serve as a novel and viable NTR procedure for POP.

## Background

Pelvic organ prolapse (POP) is the abnormal positioning of pelvic organs in which pelvic organs, such as the bladder, uterus, rectum, and vaginal wall, drop and prolapse through the vaginal opening owing to a decrease in the supporting ability of pelvic floor muscles. Lifestyle habits that cause abdominal pressure, such as aging, childbirth, intense exercise, labor, and constipation, and decrease in female hormones are reportedly involved [1–3]. These factors cause the loosening of pelvic floor muscles and endopelvic fascia, leading to the abnormal positioning of pelvic organs, often manifesting as pelvic floor hernia.

According to an epidemiological survey in the United States and a European country, 44% of parous women had POP [4] and 11% had received surgery for POP or urinary incontinence by the age of 80 years [5, 6]. A more recent epidemiological survey has reported an even higher percentage; 20% of women had received these surgeries by the age of 80 years [7]. According to a report in Japan, POP was observed in approximately 17% of women aged 21–84 years [8]. As the mean life expectancy of women has increased in recent years, this disease is expected to increase in the future. POP tends to occur among women in middle and advanced age, and it greatly deteriorates their quality of life (QOL).

The treatment of POP aims to resolve serious symptoms, such as lower urinary tract symptoms, sexual dysfunction, and a feeling of organ prolapse, and improve the QOL by correcting organ positioning. Surgical therapy is selected when pessary insertion causes complications, such as vaginal ulcers, or when QOL does not improve after conservative therapy [9]. Conventionally, anterior/posterior colporrhaphy has long been performed as a surgical intervention after vaginal total hysterectomy. However, this surgical procedure is associated with issues, such as a high recurrence rate and the lack of consideration for postoperative sexual function [5, 10–12].

With the recent introduction of mesh surgery (Tension-free vaginal mesh: TVM), surgical interventions for POP have advanced dramatically. However, the use of surgical mesh received a warning from the United States Food and Drug Administration (FDA) as it was associated with various problems, such as postoperative infection and dyspareunia owing to mesh detachment, erosion, and hardening [13]. Moreover, mesh surgery is difficult owing to risk factors, for patients who are expected to have extensive intraperitoneal adhesion and those with increased susceptibility to infection. These factors pose a high risk for developing erosion and infections as mesh-specific complications. As a result, active interest in native tissue repair (NTR) has been revived. The NTR procedures include vaginal stump–uterosacral ligament fixation (Shull method) reported by Shull et al. in which the vaginal canal axis is reconstructed without a mesh as in laparoscopic sacrocolpopexy (LSC) [14]. However, effective repair may not be achievable by simply fixing the vaginal stump and the uterosacral ligament in severe POPs in which the vaginal canal is long or when the uterosacral ligament is overextended or weakened.

To solve this problem, we have devised and reported laparoscopic vaginal stump–round ligament fixation (Kakinuma method) as a new NTR procedure for patients with POP [15]. The uterine round ligament is a histologically strong tissue positioned anatomically even higher than the uterosacral ligament. Moreover, when the uterine round ligament is overextended, adjustment can be easily made by resecting the extended round ligament so that the vaginal stump is raised 4–5 cm from the vaginal opening before it is fixed and sutured to the uterine round ligament.

This study aimed to retrospectively and clinically compare the conventional Shull method and the Kakinuma method as NTR procedures in patients with POP.

## Methods

This study was approved by the Ethics Committee of the International University of Health and Welfare (approval number: 21-B-463). Treatment was provided to all the patients after obtaining their informed consent via an adequate informed consent process. Of a total of 78 patients who underwent surgical treatment for the diagnosis of POP between January 2017 and June 2022 and follow-up observation for at least 12 months after surgery, 40 patients who underwent the Shull method (Shull group) and 38 patients who underwent the Kakinuma method (Kakinuma group) were retrospectively analyzed. Surgical outcomes were examined based on their medical records, including age, gravida and parity, comorbidities, POP-Q stage (Table 1) [16], operative duration, blood loss, intraoperative complications, and incidence of recurrence.

**Table 1:** Stages of POP–Q system measurement

Stage 0	no prolapse is demonstrated
Stage 1	the most distal portion of the prolapse is more than 1 cm above the level of the hymen
Stage 2	the most distal portion of the prolapse is 1 cm or less proximal or distal to the hymenal plane
Stage 3	the most distal portion of the prolapse protrudes more than 1 cm below the hymen but protrudes no farther than 2 cm less than the total vaginal length
Stage 4	vaginal eversion is essentially complete

At the beginning of the surgery, patients in both groups were placed in the lithotomy position under general anesthesia. Both methods were performed in a 20° head-down position using a 10-mm 0° rigid scope with a pneumoperitoneum pressure of 10 mmHg. Using the open approach method, the trocars were placed in a diamond configuration, with a 12-mm camera port at the umbilical area and 5-mm ports at three sites in the lower abdomen; the surgical trocars were positioned 3 cm medial to the anterior superior iliac spines and their midpoint. After incising the vesicouterine pouch, the round and infundibulopelvic ligaments were cut. After identifying the uterine artery and the ureter, the main trunk of the uterine artery was ligated. The bladder was separated from the cervix, and bilateral cardinal ligaments and parauterine tissues were ligated and cut. The vaginal canal was released from the posterior vaginal fornix and then circumferentially incised from the vaginal lumen along the vaginal portion of the cervix before the uterus was transvaginally removed. The vaginal stump was closed using a continuous suture with 1 − 0 absorbable thread (Ti-Cron™, Covidien Japan Co, Japan).

**Fig. 1:**
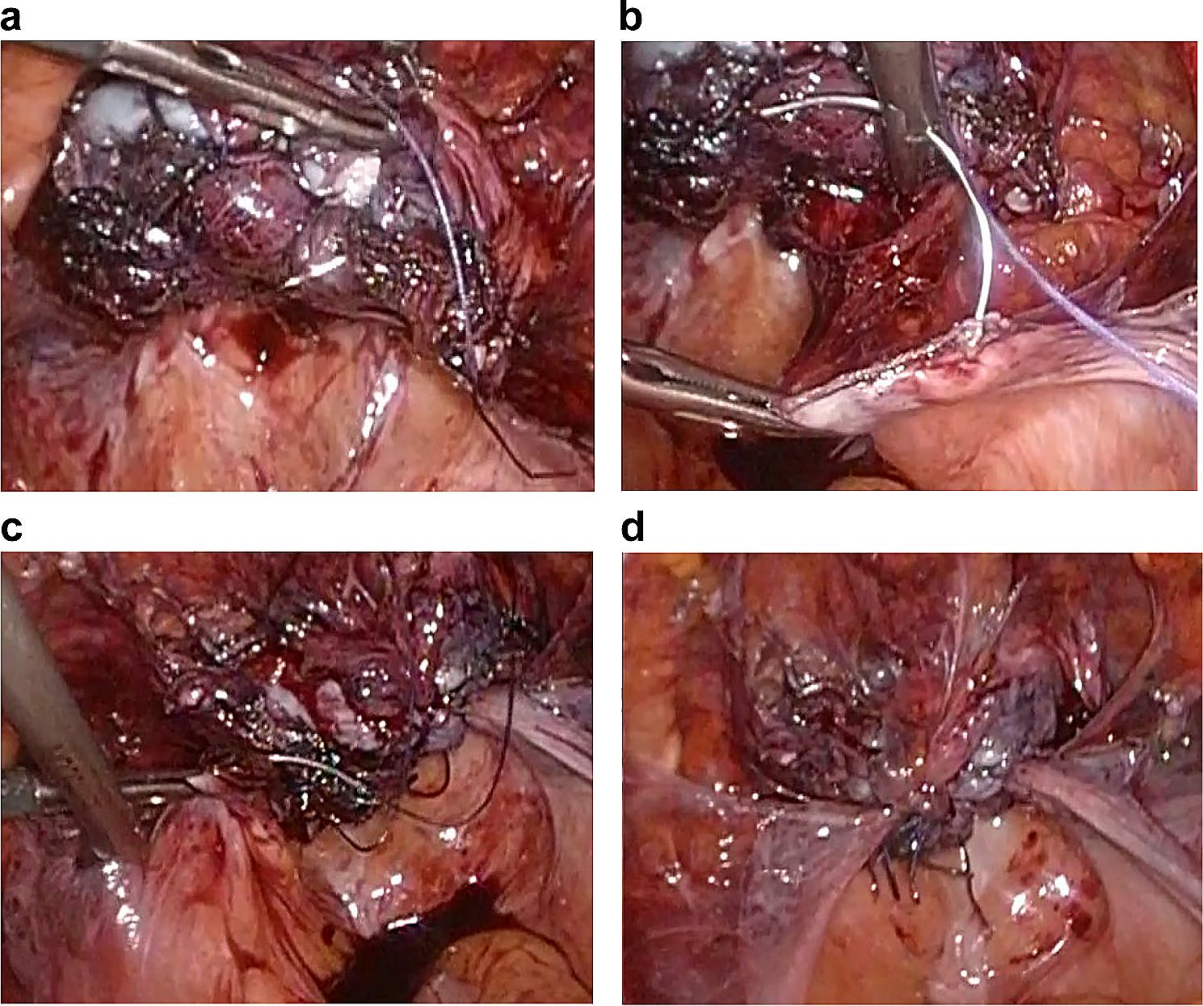
Shull method Suturing was done using 2 − 0 delayed absorbable thread by passing the needle sequentially from the anterior vaginal wall of the vaginal stump to the posterior vaginal wall and the distal end of the right uterosacral ligament (**a, b**), and suture fixation was done so that the center of the vaginal stump was placed at the center of the uterosacral ligament. Similarly, the left vaginal stump and the left uterosacral ligament were sutured and fixed using 2 − 0 delayed absorbable thread (**c, d**)

In the Shull method, after the uterosacral ligament was identified, the ureter was identified and separated by approaching along the uterosacral ligament from the lateral peritoneum to the retroperitoneal cavity. Suturing was done using 2 − 0 delayed absorbable thread (Ti-Cron™, Covidien Japan Co, Japan) by passing the needle sequentially from the anterior vaginal wall of the right vaginal stump to the posterior vaginal wall and the distal end of the right uterosacral ligament (Fig. 1a, b). Suture fixation was done so that the center of the vaginal stump was placed at the center of the uterosacral ligament. Similarly, the left vaginal stump and the left uterosacral ligament were sutured and fixed using 2 − 0 delayed absorbable thread (Ti-Cron™, Covidien Japan Co, Japan) (Fig. 1c, d).

**Fig. 2:**
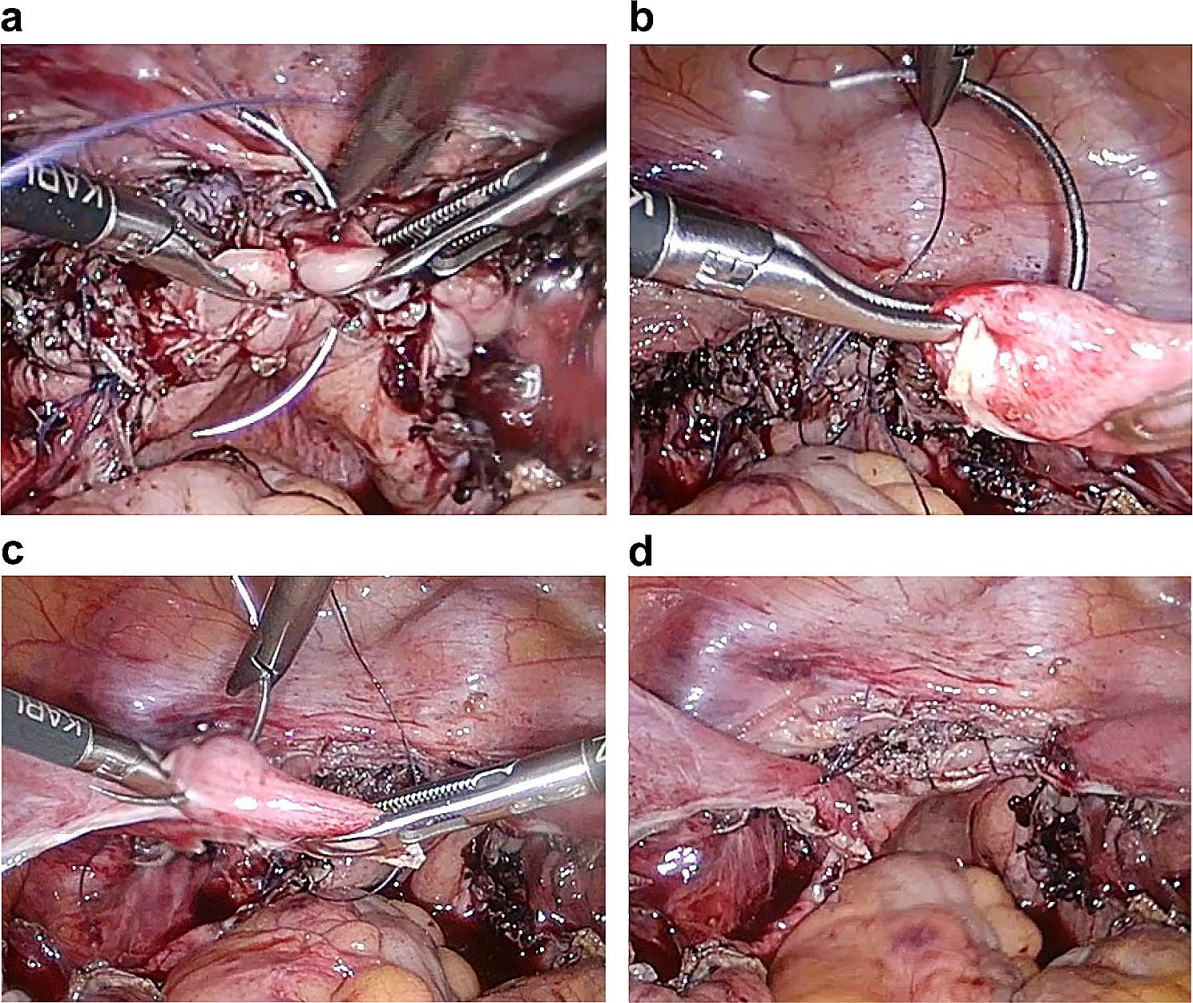
Kakinuma method Suturing was done using 2 − 0 delayed absorbable thread by passing the needle sequentially from the anterior vaginal wall of the vaginal stump to the posterior vaginal wall and the distal end of the right uterine round ligament (**a, b**). The center of the vaginal stump and the proximal end of the uterine round ligament were sutured and fixed so that the vaginal stump was positioned 4–5 cm from the vaginal opening. Similarly, the left vaginal stump and the left uterine round ligament were sutured and fixed using 2 − 0 delayed absorbable thread (**c, d**)

In the Kakinuma method, suturing was done using 2 − 0 delayed absorbable thread (Ti-Cron™, Covidien Japan Co, Japan) by passing the needle sequentially from the anterior vaginal wall of the right vaginal stump to the posterior vaginal wall and the distal end of the right uterine round ligament (Fig. 2a, b). Suture fixation was done so that the center of the vaginal stump was placed at the center of the uterine round ligament. Similarly, the left vaginal stump and the left uterine round ligament were sutured and fixed using 2 − 0 delayed absorbable thread (Ti-Cron™, Covidien Japan Co, Japan) (Fig. 2c, d). In cases where the round ligaments were overextended, the redundant round ligaments were shortened via resection so that the vaginal stump was positioned 4–5 cm from the vaginal opening, and the vaginal stump was sutured to the round ligament.

In both groups, after vaginal stump fixation, the retroperitoneum was closed by continuous suturing. After confirming that there was no bleeding in the abdominal cavity, an adhesion-preventing agent (AdSpray®, Terumo Corporation, Tokyo, Japan) was sprayed, and the surgery was completed.

In statistical analysis, continuous variables were compared using the Mann–Whitney U test and categorical variables were compared using the Fisher’s exact test. *P* < 0.05 was considered to indicate that the difference was statistically significant.

## Results

A total of 78 cases were included, comprising 40 cases in which the Shull method was used (Shull group) and 38 cases in which the Kakinuma method was used (Kakinuma group). Patient background data are shown in Table 2. No significant differences were observed between the two groups in patient background data, such as the mean age, parity, BMI, and POP-Q stage. Surgical outcomes are shown in Table 3. The mean operative duration and mean blood loss in the Shull group were 140.5 ± 31.7 min and 91.3 ± 96.3 ml, respectively, whereas the respective values in the Kakinuma group were 112.2 ± 25.3 min and 31.4 ± 47.9 ml, respectively. Thus, the Kakinuma procedure was completed in a significantly shorter time (*P* < 0.001) and was associated with a smaller bleeding volume (*P* = 0.003) compared with the Shull procedure. The postoperative observation period was 23.5 ± 8.0 months in the Shull group and 23.4 ± 7.3 months in the Kakinuma group, with no significant difference (*P* = 0.964). Recurrence of POP was observed in six patients (15.0%) in the Shull group and two patients (5.3%) in the Kakinuma group. Hence, recurrence was significantly less frequent in the latter group (*P* = 0.015). No perioperative complications were observed in either group.

**Table 2:** Patient Background

	Shull method (*N* = 40)	Kakinuma method (*N* = 38)	*P* values
Age (years)	70.3 ± 8.0	67.1 ± 9.4	0.11
BMI	24.6 ± 2.7	25.0 ± 3.0	0.46
Parity (time)	2.3 ± 0.8	2.5 ± 0.7	0.21
POP-Q stage (case)			
II	17	13	
III	17	17	
IV	6	8	

**Table 3:** Surgical Outcomes

	Shull method (*N* = 40)	Kakinuma method (*N* = 38)	*P* values
Operative duration (min)	140.5 ± 31.7	112.2 ± 25.3	*P* < 0.001
Blood loss (ml)	91.3 ± 96.3	31.4 ± 47.9	0.003
Recurrence (case)	6	2	0.015

## Discussion

This study compared laparoscopic vaginal stump–round ligament fixation (Kakinuma method) as a new NTR for POP with the Shull method as a conventional NTR in terms of usefulness and safety. The treatment of POP aims to mitigate symptoms by anatomically restoring the abnormal positioning of the prolapsed organs. Conservative therapies include physical methods, such as pelvic floor muscle exercises, and noninvasive recovery methods using pessaries. However, for moderate to severe POP, surgical therapy must be selected. Most patients with this disease are older people and have decreased overall functional capacities, such as decreased organ reserve, wound healing, and immune capacities, as well as comorbidities. Thus, the procedure for surgical therapy should be selected based on patient background factors, such as general condition.

Conventionally, vaginal total hysterectomy and colporrhaphy have been widely performed as surgical therapies for POP. However, it is difficult to repair POP by simply dissecting excess mucous membrane of the vaginal wall and suture repairing, and the recurrence rate has been reported to be high [5, 10–12].

Subsequently, the tension-free vaginal mesh (TVM) procedure in which a mesh is used to replace the pubocervical fascia and rectovaginal fascia, which are the most important supporting structures in the pelvic floor, has been reported and used mainly in the field of urology [17]. This surgery, which was introduced in the United States and European countries in 2000, restores the dropped uterus and bladder to the original position and reinforces them using a polypropylene mesh specifically for POP surgery. TVM gained popularity because of reduced postoperative pain and low recurrence rate. However, FDA issued a warning because it caused serious complications, such as mesh exposure to the vagina and bladder/rectal injuries [13], and mesh for TVM surgery to treat POP was discontinued in 2012.

Meanwhile, a surgical procedure that raises the vaginal stump to the promontorium abdominally was reported in 1957, and with the subsequent widespread use of the mesh, abdominal sacrocolpopexy was developed. However, it was not commonly used because the procedure was highly invasive, and intraoperative complications, such as major bleeding from the sacrum, were reported [18, 19]. However, with the development of laparoscopic techniques, LSC is becoming popular as it is less invasive and has shown therapeutic effectiveness comparable to that of laparotomy [20]. However, there are many cases in which mesh surgery is difficult to select because of risk factors, including patients expected to have severe intraperitoneal adhesion and, as mentioned above, patients at a high risk of developing erosion and infection owing to increased susceptibility to infection. For these reasons, NTR has regained the importance in the treatment of POP [21, 22]. The standard surgical procedure of NTR is based on the vaginal technique, and vaginal stump fixation is performed after total hysterectomy. Vaginal stump fixation methods include the Shull method in which the stump is fixed to the uterosacral ligament [14]. This method is characterized by the elevation of the vaginal canal in a physiological direction; however, there is a risk of ureteral injury or obstruction as the ureter is in close proximity to the uterosacral ligament. Moreover, effective repair may not be achievable by simply fixing the vaginal stump and the uterosacral ligament in patients with severe POP because the vaginal canal is long and in patients with an overextended or weakened uterosacral ligament. Therefore, to solve these problems, we devised the Kakinuma method as a safe and effective NTR procedure. In this surgical procedure, the vaginal stump is fixed to the uterine round ligament, which is anatomically higher and histologically stronger and has no vital organs in the adjacent areas, after laparoscopic total hysterectomy [17, 23]. Historically, POP surgery has been performed transvaginally; however, it is not easy to acquire sufficient skills to perform this procedure owing to the narrow visual field. The use of laparoscopy allows the sharing of a clear, magnified visual field with the surgical staff. Moreover, as laparoscopic total hysterectomy has recently become a basic surgical procedure for obstetricians and gynecologists, no new special surgical equipment or tool is required. Furthermore, the procedure is not so technically demanding and has minimal effects from surgeons’ skill levels because there are no important organs around the uterine round ligament. In this study, no surgical complications were observed in the cases analyzed. Another advantage is that this surgical procedure has minimal intraoperative complication risks, such as organ injuries during retroperitoneal tunnel creation for mesh insertion and vascular injuries during fixation of the mesh to the anterior longitudinal ligament of the sacral promontory in LSC [24–26]. When it is desirable to avoid LSC for reasons such as adhesion, it is possible to immediately switch to this method.

In patients with an overextended uterine round ligament, the vaginal stump can be effectively lifted by fixing the vaginal stump after resecting the redundant uterine round ligament so that it is positioned 4–5 cm from the vaginal opening.

In this study, the Kakinuma method for POP was completed in a significantly shorter time with a smaller bleeding volume and a lower recurrence rate compared with the conventional Shull method. The results suggest that it could be a new NTR procedure for POP. However, a limitation of this surgical procedure is that this procedure alone may not suffice. Its use as a hybrid method with conventional transvaginal colporrhaphy and perineoplasty, such as levatorplasty, may be required when further reinforcement of the posterior vaginal wall is needed or if the patients have cystocele or rectocele.

This study included patients who underwent follow-up observation for at least 12 months after the surgery. This was a single-center retrospective study in a small number of patients. In the future, it is necessary to accumulate more cases and perform follow-up evaluation for a longer term. Therefore, it is important to evaluate cases in detail from various aspects, such as recurrence and late postoperative complications, and determine the criteria of indication for this surgical procedure.

## Conclusions

Treatment of POP using the Kakinuma method was completed in a significantly shorter time with a smaller bleeding volume and a lower recurrence rate compared with the conventional Shull method. The results shown here suggest that this surgical procedure could be a new option of NTR for POP.

## Data Availability

All data generated or analyzed during this study are included in this article. Further inquiries can be directed to the corresponding author at tokakinuma@gmail.com.

## Abbreviations

FDA: Food and Drug Administration
NTR: Native tissue repair
POP: Pelvic organ prolapse
QOL: Quality of life
TAH: Total abdominal hysterectomy
TLH: Total laparoscopic hysterectomy
TVM: Tension-free vaginal mesh
